# Computational Approaches for Acute Traumatic Brain Injury Image Recognition

**DOI:** 10.3389/fneur.2022.791816

**Published:** 2022-03-09

**Authors:** Emily Lin, Esther L. Yuh

**Affiliations:** Department of Radiology and Biomedical Imaging, University of California, San Francisco, San Francisco, CA, United States

**Keywords:** traumatic brain injury, deep learning, artificial intelligence, precision medicine, evidence-based medicine, image recognition

## Abstract

In recent years, there have been major advances in deep learning algorithms for image recognition in traumatic brain injury (TBI). Interest in this area has increased due to the potential for greater objectivity, reduced interpretation times and, ultimately, higher accuracy. Triage algorithms that can re-order radiological reading queues have been developed, using classification to prioritize exams with suspected critical findings. Localization models move a step further to capture more granular information such as the location and, in some cases, size and subtype, of intracranial hematomas that could aid in neurosurgical management decisions. In addition to the potential to improve the clinical management of TBI patients, the use of algorithms for the interpretation of medical images may play a transformative role in enabling the integration of medical images into precision medicine. Acute TBI is one practical example that can illustrate the application of deep learning to medical imaging. This review provides an overview of computational approaches that have been proposed for the detection and characterization of acute TBI imaging abnormalities, including intracranial hemorrhage, skull fractures, intracranial mass effect, and stroke.

## Introduction

Acute traumatic brain injury (TBI), defined as sudden physical trauma that results in damage to the brain, is diagnosed through clinical assessment, with considerable reliance on structural neuroimaging studies such as computed tomography (CT) and occasionally, magnetic resonance imaging (MRI) (1). CT is the primary imaging modality worldwide for diagnosis of acute TBI due to its high sensitivity for acute intracranial hemorrhage and skull and facial fractures, rapid scan times, and lack of absolute contraindications (2, 3). While MRI is more sensitive to certain pathoanatomic features of acute TBI such as traumatic axonal injury and small cortical contusions, it is not routinely performed on acute TBI patients, but may be used as a follow-up study in patients with persistent or unexplained neurological deficits (4, 5). The rapid and accurate interpretation of these structural imaging studies in acute TBI is critical; imaging informs immediate clinical decisions such as hospitalization, intensive care unit admissions, and neurosurgical intervention, and also bears prognostic information. Structural imaging studies are interpreted by radiologists, but there has also been increasing interest in the development of image recognition algorithms that can aid, augment, and streamline the image interpretation process.

The use of algorithms for the detection of acute TBI pathoanatomic features has several key advantages. Automated algorithms could reduce the time to diagnosis and treatment and help improve patient outcomes. They also have the potential to extract useful information from medical imaging studies in a quantitative and objective manner, which can greatly facilitate the integration of diagnostic medical imaging with advances in precision and evidence-based medicine. Radiological interpretations by human observers, in contrast, are subjective and vary based on differences in experience and judgment. In addition to this, algorithms are not affected by fatigue or “satisfaction of search,” a very common problem in which detection of one radiographic abnormality causes a human observer to overlook an additional critical, but unexpected, abnormality (6). In order for algorithmic approaches to be of clinical relevance, however, they must demonstrate very high accuracy levels on large datasets that are representative of the population.

Early attempts to develop algorithms to interpret head CT exams used rule-based or traditional machine learning approaches. For the former, researchers would define a set of heuristics for the detection of pathoanatomic features (7–10). Despite a considerable amount of painstaking manual tuning through trial and error and promising preliminary results, however, it was not possible to curate a set of rules that could distinguish abnormal from normal images with accuracies in the range of those needed for clinical utility. Machine learning approaches, consisting of models that detected lesions by learning directly on a set of training examples, were also used. These approaches were attractive because they reduced the need for painstaking trial and error and pre-programming. Studies reported a variety of machine learning strategies for head CT interpretation, including random forest (11, 12), support vector machine (13), and decision tree (14–16). However, the rigid analytic forms of traditional machine learning models still made it difficult to accurately model complex imaging data. Ultimately, neither rule-based nor machine learning strategies were sufficient to achieve the strong performance levels needed for clinical use.

Just over 10 years ago, advances in computer hardware enabled a 100-fold acceleration of matrix computations that are fundamental to neural networks, a class of machine learning algorithms (17). Reduced data storage costs also made it easier to amass large datasets. These developments made it feasible, for the first time, to perform massive numbers of matrix manipulation tasks on enormous datasets in a reasonable amount of time, and led to a resurgence of interest in deep neural networks, or “deep learning.” Unlike traditional machine learning models, which are fixed in analytic form, deep learning models are flexible, have potential for essentially limitless complexity, and can theoretically model any arbitrary mathematical function (18). In real-world applications, the required quantity of training data, which roughly scales with the depth of a deep learning model, does impose a practical limit on the depth and complexity of a deep learning model. Nevertheless, the increased representation power, elimination of the need for manual tuning, and more efficient large-scale learning have resulted in profound increases in accuracy in image recognition tasks. As a result, deep learning has rapidly dominated the computer vision field in the last decade, with advances also diffusing into the medical field. Examples of early demonstrations of deep learning applied to medical imaging include detection of pneumonia on chest x-ray (19) and tuberculosis (20) and lymph node metastases (21) and non-small cell lung cancer (22) on histopathology slides.

Deep learning models have also been successfully developed for the analysis of neuroimaging studies in acute TBI. Numerous triage algorithms have been developed for the automated classification of imaging abnormalities, with the goal of future implementation into the radiological workflow to decrease time to diagnosis (23–26). Simultaneously, localization algorithms were also being developed and trained to automatically segment abnormality boundaries to extract granular information such as lesion size, subtype, number, and location (27–29). This review will provide an overview of computational approaches for the detection of TBI imaging abnormalities, including intracranial hemorrhage, fractures, mass effect, and stroke.

## Methods

A targeted literature review was performed using Google Scholar to identify publications related to computational approaches for image recognition in acute TBI. Search queries included combinations of the following keywords: “Traumatic brain injury,” “Deep learning,” “Machine learning,” “Automated detection,” “Head computed tomography,” “Magnetic Resonance Imaging,” “Intracranial hemorrhage,” “Skull fracture,” “Intracranial mass effect,” “Edema,” and “Stroke” without any restrictions on publication year. Articles that were most relevant to the review topic were selected, with an effort to identify and include all key articles with major contributions to the field.

## Traumatic Brain Injury

Traumatic brain injuries can be classified either as penetrating, in which a foreign body traverses the skull and enters the intracranial space, or non-penetrating (also known as closed head injury or blunt TBI). TBI is divided into three clinical severity categories: mild, moderate and severe. The primary criterion that determines clinical severity category is the Glasgow Coma Scale (GCS) score. Patients presenting with GCS scores from 3 to 8, 9 to 12, and 13 to 15 are classified as having severe, moderate, and mild TBI respectively, with mild TBI accounting for ~90% of acute TBI patients (30). In some grading schemes, mild TBI classification also requires that the patient exhibit no focal neurological deficit on initial examination (31, 32). Although clinical severity has long been based primarily on the GCS score, this coarse classification belies considerable heterogeneity in the underlying neuropathology and clinical outcomes of patients with identical GCS scores (33, 34). The current consensus is that development of more granular TBI classification schemes is a critical need in order to develop effective therapies for acute TBI (34).

### Computed Tomography (CT)

With rare exception, head CT is the imaging modality used to evaluate patients with suspected acute TBI. A small proportion of patients in the Emergency Department (ED) are judged to have sustained very mild head injuries based on a benign mechanism of injury, presenting GCS score of 15, and no loss of consciousness or posttraumatic amnesia, and may be discharged from the ED without head imaging. For all others, non-contrast head CT is performed (32). Over 20 million head CT scans are performed annually in the U.S. Head CT is the near universal choice for initial imaging in acute TBI in infants, children and adults, due to its very high sensitivity for acute intracranial hemorrhage and skull and facial fractures, widespread 24-h availability (35), and lack of any absolute contraindication. In addition, it's extremely short acquisition time, which allows it to perform whole-head imaging in as little as 0.3 s using modern multidetector-row CT scanners (36), is essential in many cases in which patients are unable to remain still due to altered mental status, pain, or young age.

**Figure 1:**
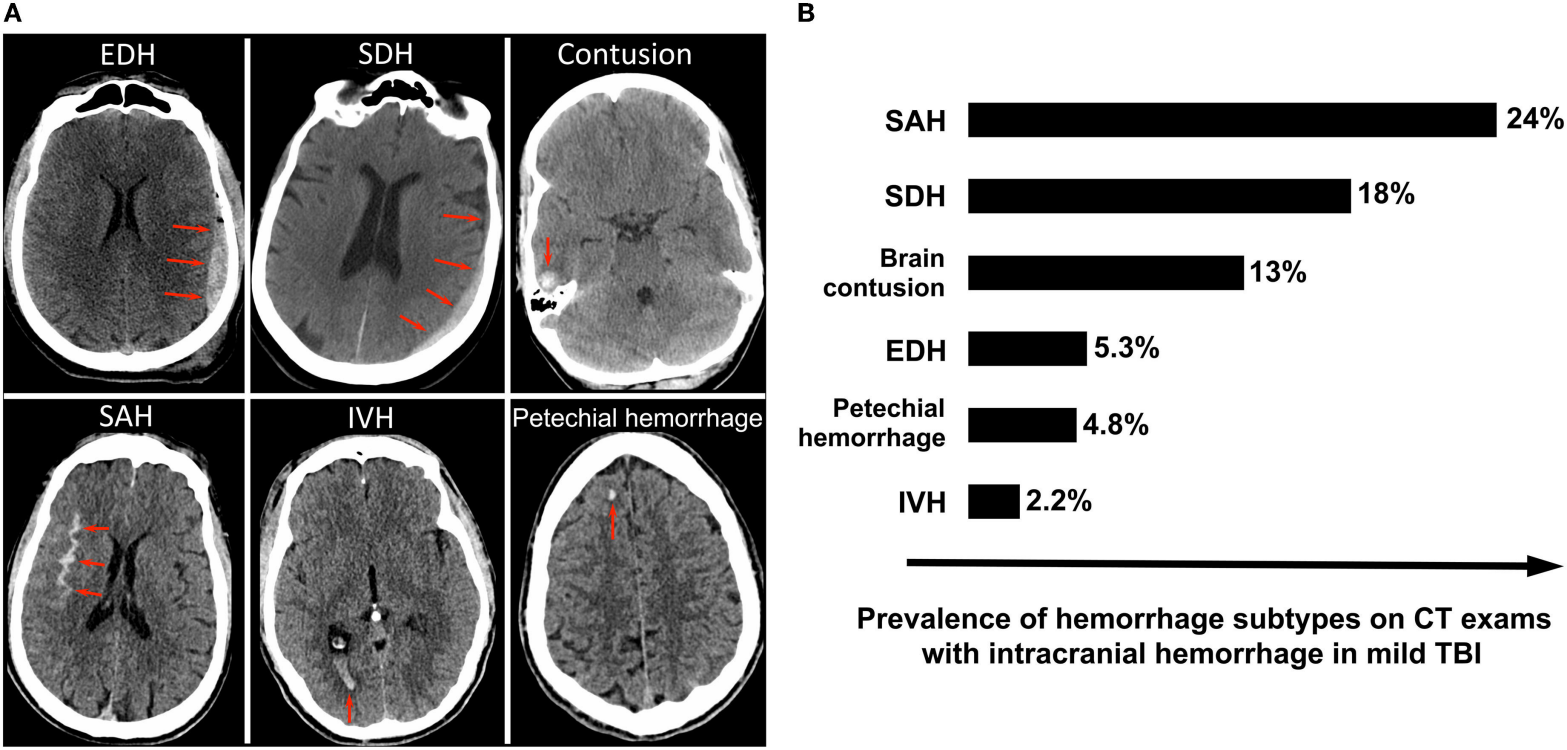
Intracranial hemorrhage subtypes and their frequencies among mild TBI patients enrolled in the TRACK-TBI prospective longitudinal study of acute TBI (39). **(A)** Illustrates the various subtypes of intracranial hemorrhage, with red arrows indicating the abnormal lesion. **(B)** Shows the frequencies of each subtype of hemorrhage. SAH is the most commonly observed subtype, followed by SDH and contusion. Although the overall average incidence of “complicated” mild TBI (mild TBI with presence of acute intracranial hemorrhage on head CT) in the U.S. is lower in clinical practice than in TRACK-TBI (40), the relative distribution of hemorrhage subtypes within mild TBI is likely similar (39).

Approximately 9% of head CT scans on acute TBI patients demonstrate acute intracranial hemorrhage (Korley, 2016). GCS score is a significant predictor of intracranial hemorrhage, identified on ~9% of CT scans in mild TBI (37), 56% in moderate TBI, and 81% in severe TBI (38). Subtypes of acute intracranial hemorrhage include epidural hematoma (EDH), subdural hematoma (SDH), contusion, subarachnoid hemorrhage (SAH), intraventricular hemorrhage (IVH), and petechial hemorrhage (Figure 1A). The most common subtypes are SAH, SDH and brain contusion (Figure 1B). Although there is a correlation between the initial GCS score and the presence of intracranial hemorrhage, all subtypes of intracranial hemorrhage are observed at all clinical severity levels. For example, SAH is the most common subtype of intracranial hemorrhage across all severity levels, although it is observed in 24.4% of mild TBI patients and 43% of moderate and severe TBI patients (38, 39). Skull fractures are also seen across all GCS scores, although are much more common in severe TBI [~47% (41)] compared to mild TBI [~3% (42)]. While midline shift and other types of brain herniation are also present in all severity categories, they are much more common in moderate and severe TBI, affecting ~60% of these patients (43) compared to 3% of mild TBI patients (44). Lastly, while ischemic stroke does occasionally occur in acute TBI patients, it is rare and occurs in ~2.5% of moderate and severe TBI (45). While a brief discussion of deep learning applications for stroke is included in this review, it is usually treated as a distinct disorder.

### Brain MRI

Brain MRI, including basic structural (anatomic) brain MRI, is not currently recommended as the initial diagnostic imaging modality for acute TBI in adults (46, 47), children or infants (48). Brain MRI protocols for evaluation of acute TBI generally include T1-weighted, T2-weighted, T2-weighted Fluid Attenuated Inversion Recovery (FLAIR) sequences, diffusion-weighted imaging (DWI), and either susceptibility-weighted imaging (SWI) or T2^*^-weighted gradient echo (GRE) (49). MRI is highly sensitive to certain traumatic intracranial findings such as traumatic/diffuse axonal injury, small cortical contusions, and small extra-axial collections (50, 51), and is occasionally performed in hospitalized TBI patients as a problem solving tool in cases when the level of consciousness is persistently impaired and not accounted for by findings on initial head CT. Brain MRI is also used to evaluate persistently symptomatic patients in the subacute or chronic stages after TBI, in medico-legal cases, and in cases of suspected abusive head trauma to assess for evidence of brain injuries of different ages (52).

The use of more advanced MRI techniques such as diffusion tensor imaging (DTI) and functional magnetic resonance imaging (fMRI) in acute TBI are highly promising techniques for more detailed and nuanced characterization of damage to the brain in TBI (49, 53). DTI in particular has been shown in numerous studies to demonstrate significant group differences in physical characteristics of the white matter tracts between acute TBI and control patients (54–56) that are also correlated with patient outcome. While most studies to date of DTI for acute or post-acute TBI have demonstrated group differences between TBI and control patients, there is not yet a consensus on how these techniques should be used clinically for diagnosis of individual acute TBI patients. Unlike CT and “basic” structural brain MRI, DTI, and fMRI data are not directly evaluated through human visual interpretation, but are inherently quantitative studies that undergo extensive post-processing using traditional statistical and machine learning techniques (57), and are not currently recommended for clinical use (46, 47). Interpretation of these studies is largely based on what the data show relative to “statistical significance” threshold values, rather than on subjective human visual inspection. Although machine learning, including deep learning, will likely have an increasing role in the analysis of DTI and fMRI data as these techniques continue to mature, these topics are beyond the scope of the current focused review.

## Overview of Algorithms for Computer Analysis of Medical Imaging

### Rule-Based Algorithms

The term “computer-aided diagnosis” (CAD) has existed in the scientific literature for ~50 years (58). For nearly all of that time, researchers have proposed heuristic, or rule-based, approaches for the analysis of neuroimaging studies in acute TBI. Rule-based approaches for image analysis use a set of “hand-crafted” rules created by a human observer based on morphology, brightness, and other characteristics of an image or imaging feature that can be perceived by the human visual system. Examples of such rule-based approaches, e.g., for acute intracranial hemorrhage detection on head computed tomography (CT), include algorithms based on top-hat transformation and left-right asymmetry (7); thresholding and connectivity (8); thresholding, linear and circular Hough transformations, and cluster analysis (9), and fuzzy c-means clustering (FCM) and region-based active contour (10). Though these and other rule-based approaches demonstrated the feasibility of using CAD to extract useful information from head CT images, they generally fell far short of accuracy levels that would be needed to make these approaches clinically useful. Reported performances either remained far below the threshold needed for clinical utility or were tested on CT exams that were not randomly selected.

**Figure 2:**
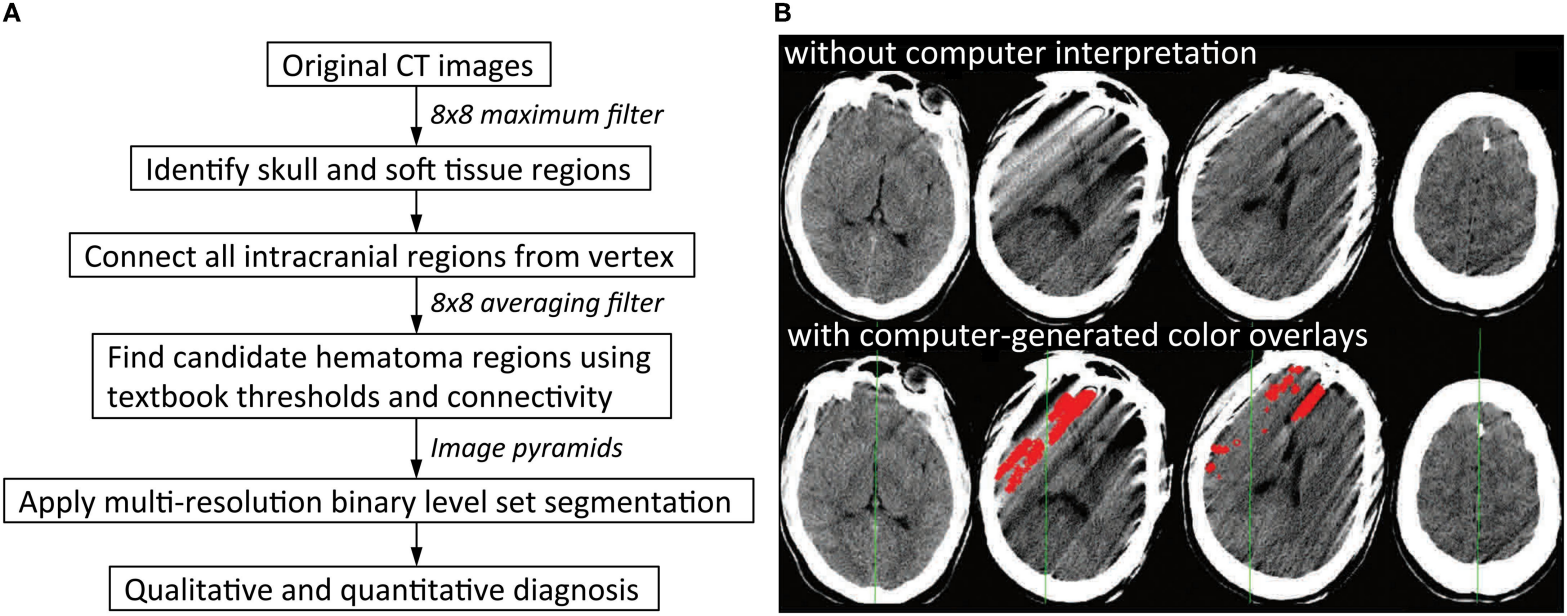
Rule-based algorithms. **(A)** Illustrates one possible workflow for a rule-based intracranial hemorrhage detection model. Application of thresholding and connectivity is a common way to identify regions of hematoma (8). **(B)** Shows examples of false positive errors from rule-based models in which streak artifacts are incorrectly labeled as regions of intracranial hemorrhage. This can result if regions of hemorrhage are obtained by thresholding for brighter pixels, which is a common strategy in rule-based models. Red indicates regions of algorithmic predictions (9).

Rule-based strategies are limited because it is challenging to enumerate all the rules that would fully describe the diversity in appearance of normal anatomy as well as pathological imaging findings seen in clinical practice. This problem is compounded by a large number of potential technical artifacts that are easily recognized by human observers, but have highly variable imaging appearances that are difficult to codify reliably. For instance, thresholding for brighter areas is a common rule-based strategy to identity acute intracranial hemorrhage, because acute hemorrhage is characterized by CT densities from 50 to 100 Hounsfield units and present as hyperdense, or “bright,” regions on head CT images (Figure 2A). However, Figure 2B shows an example in which a rule-based algorithm employing thresholding, cluster analysis, and morphological analysis incorrectly labels streak artifacts as acute ICH due to its brightness.

### Traditional Machine Learning Algorithms

Machine learning, a subset of artificial intelligence, seeks to minimize the reliance on heuristics and manual pre-programming and make predictions based on learned experience instead. Classical machine learning algorithms are broadly divided into supervised and unsupervised models. In supervised learning, the model receives as input both the data and the desired output, and “learns” from training data such that its predictions match the desired output as closely as possible. The use of labeled training data (with labeling performed by human experts and/or other computer algorithms) often results in stronger performances, making supervised learning the paradigm of choice for most practical applications including medical imaging. In contrast, unsupervised learning models receive input data with no labels and learn by identifying the underlying structural patterns or groupings in the data itself. Depending on the algorithm choice, model training is an iterative process in which the algorithm periodically updates its own parameters to better approximate the desired function and minimize the error in its output predictions. Numerous machine learning algorithms, including logistic regression (12), random forest (11, 12, 59), Bayesian decision theory (60), k-means clustering (61), and support vector machines (13, 62), have been used for the detection and segmentation of acute TBI imaging features. Each of these is briefly described below.

Logistic regression produces a binary output based on independent predictors (63). The random forest algorithm is an ensemble learning strategy that leverages multiple decision trees to make a classification prediction (64). Bayesian decision theory quantifies the tradeoffs between multiple choices in a probabilistic manner (65, 66). K-means clustering divides data into k groupings, where each data point is associated with the most similar group (67). The support vector machine maps input data onto a higher-dimensional grid and identifies the hyperplane that best differentiates each class, and can be more effective on complex and higher-dimensional data (68). Even though machine learning techniques such as these have considerable overlap with statistical modeling, they often differ in objectives. The goal in machine learning is usually to make accurate predictions, while the goal in statistics is often to uncover significant relationships between variables that allows broad interpretation of the data (69).

While traditional machine learning algorithms are more adaptive than rule-based methods, there remain limitations. The use of traditional machine learning for medical imaging tasks still generally requires explicit feature extraction, usually as one or more early steps in the algorithm. Such features include but are not limited to voxel CT density thresholding (12), properties of surrounding voxels (11), and shape features (13), with predictions often made based on these extracted lesion predictors rather than the input image itself. This means that traditional machine learning models are rarely applied end-to-end on raw input image pixels, which could also make it more difficult to scale the algorithm to large complex datasets. Lastly, they are limited in the functions they are able to model. Logistic regression, for example, is optimal when the data can be linearly separated. However, medical imaging data is much more complex and often cannot be modeled in a linear fashion. As a result, many of these traditional machine learning techniques are more frequently used and better suited for applications such as TBI outcome prediction (70, 71).

### Deep Learning Algorithms

The feasibility of a large-scale data-driven approach for image classification is exemplified by deep learning (72). Deep learning circumvents many of the limitations of rule-based and traditional machine learning algorithms, including heavy reliance on painstaking handcrafted rules and feature selection. It also allows “learning” of image patterns directly from large volumes of data, making it a scalable method to more efficiently generalize to external datasets. This has led to substantial boosts in accuracy of machine learning algorithms for image recognition tasks, and a paradigm shift in the field of computer vision. Deep learning underpins all current state-of-the-art algorithms for widely-benchmarked computer vision tasks such as image classification and object detection (73–77). While deep learning originated from the concept of the artificial neural network (ANN), it has since progressed to much deeper and more advanced networks including convolutional neural networks (CNNs), recurrent neural networks (RNNs), and, more recently, transformers. This section will provide an overview of the major deep learning algorithms and how they have been applied to medical imaging.

**Figure 3:**
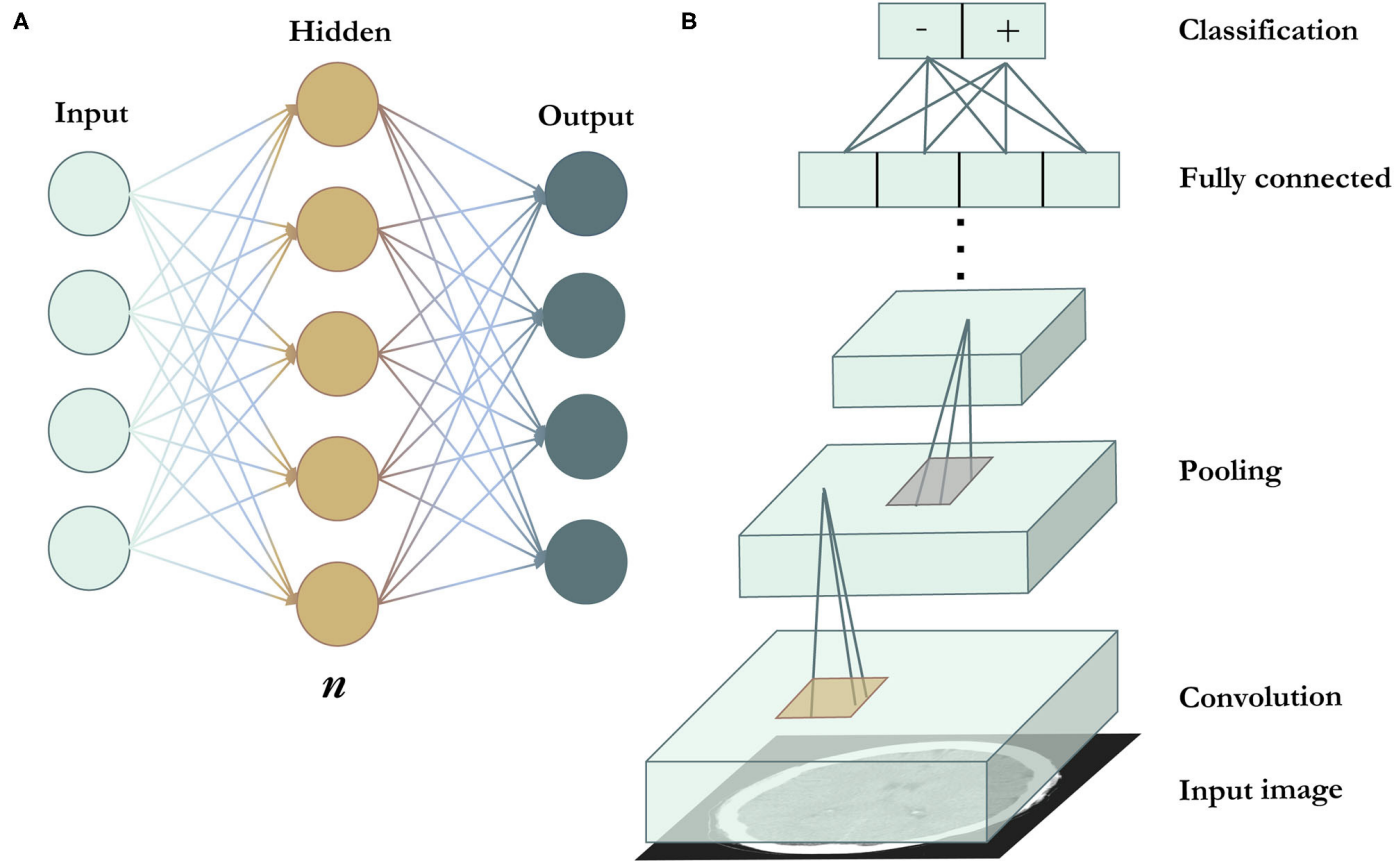
Deep learning algorithms. **(A)** Is a schematic representation of an artificial neural network. *n* indicates the number of hidden layers. A network with *n* = 1, as seen in the figure, is the most basic and shallow form of a neural network. *n* can be increased to deepen the network and broaden its representation capacity. Increasing *n* results in the ability of the network to model tasks of increasing complexity, but also requires more training data to avoid overfitting (in which the model merely exploits the extra variables to achieve high performance on a specific training dataset, but fails to perform similarly on data outside of the training dataset). **(B)** Illustrates a convolutional neural network. Convolutional filters (in gold) are applied across the image to extract features, followed by pooling filters (in silver) that reduce feature map dimensionality. Most CNNs use multiple convolutional and pooling layers. The end layers include the fully connected and output layers.

The earliest artificial neural networks (ANNs) were inspired by the transmission of information between the neurons in the human brain (78–81). The ANN includes an input layer, an output layer, and at least one “hidden” layer, with each layer designed as an array of neuron-like nodes (Figure 3A) (82, 83). The input layer accepts data, and the output layer produces a prediction. Between the input and output layers are the hidden layers, each of which accepts the output from the previous layer's nodes and performs forward computations to produce its own output. Although ANNs were highly novel at the time, they have limited representation power. This can be solved in part by increasing the numbers of layers and deepening the neural network, which increases the power of the network to “learn” subtle differences in complex patterns, because deeper networks have larger numbers of values that can be adjusted in order to model complex data. However, the simple network architecture still limited the performance of basic neural networks on more complex tasks such as visual recognition.

This problem was addressed by the development of the convolutional neural network (CNN). In 2012, a CNN called AlexNet was the winner of the ImageNet Large Scale Visual Recognition Competition (ILSVRC) (74). As one of the earliest CNNs, AlexNet used convolutional layers to detect local features, with early layers detecting simple features such as edges, corners, and colors, and higher layers detecting higher-level features such as objects (84). Filters that perform the convolution operation are applied across the input image, producing feature maps that preserve spatial invariance (Figure 3B). CNNs also apply pooling layers to reduce the dimensionality of feature maps and increase computational efficiency while retaining important image features. The CNN architecture continued to improve significantly in the subsequent years, with increased network depths and added features such as multi-scale filters (85), factorized convolutions (86), and residual connections (87). Coupled with greater computational efficiency, the CNN has since become the standard network architecture of choice for most visual tasks and has been applied to medical image analysis, including the detection (23, 25, 26) and localization of intracranial hemorrhage (27–29).

Aside from the CNN, other deep learning models have been applied to sequential data such as language, audio, and video tasks. Algorithms such as RNNs (88) and long short-term memory (LSTM) (89) augment a model with sequential memory to model temporal relationships, but often struggle to retain information when encountered with longer sequences. Transformers were recently proposed to address this limitation; they have shown great success in modeling sequential data by capturing long-range pairwise relationships and have since replaced RNNs in most practical applications (90–92). Because RNNs are best suited for sequential data, they are more commonly used for the analysis of electronic medical records and clinical reports (93, 94) rather than medical images. However, there has been recent interest in the use of transformers in medical imaging (95–97). This area is still relatively new and unexplored, but may be a promising direction in coming years.

Overall, deep learning models have benefited tremendously from recent breakthroughs in computational resources (98–100), data volume (101–103), and algorithmic advancements (74, 87, 90, 104, 105). The large number of free parameters associated with hidden layers has enabled a vastly more flexible algorithm that can model complex data and excel over rule-based and traditional machine learning approaches for image recognition tasks. Coupled with greater data availability, deep learning has facilitated the development of acute TBI imaging algorithms that are more accurate than previously seen.

## Data Labeling Strategies

Deep learning algorithms learn by gleaning patterns from large volumes of data, but they require high quality labeled data to do so. The granularity of the labels determines the maximum granularity, or level of detail, of the model's output prediction, and also affects the final accuracy of the model for a given task. Therefore, the labeling strategy partly determines the clinical applications the model may be useful for. Because deep learning models' accuracy is critically dependent on the quality and type of data they learn from, it may be useful to discuss and compare common data labeling strategies. We discuss below several common data labeling strategies, in order of increasing labeling granularity.

**Figure 4:**
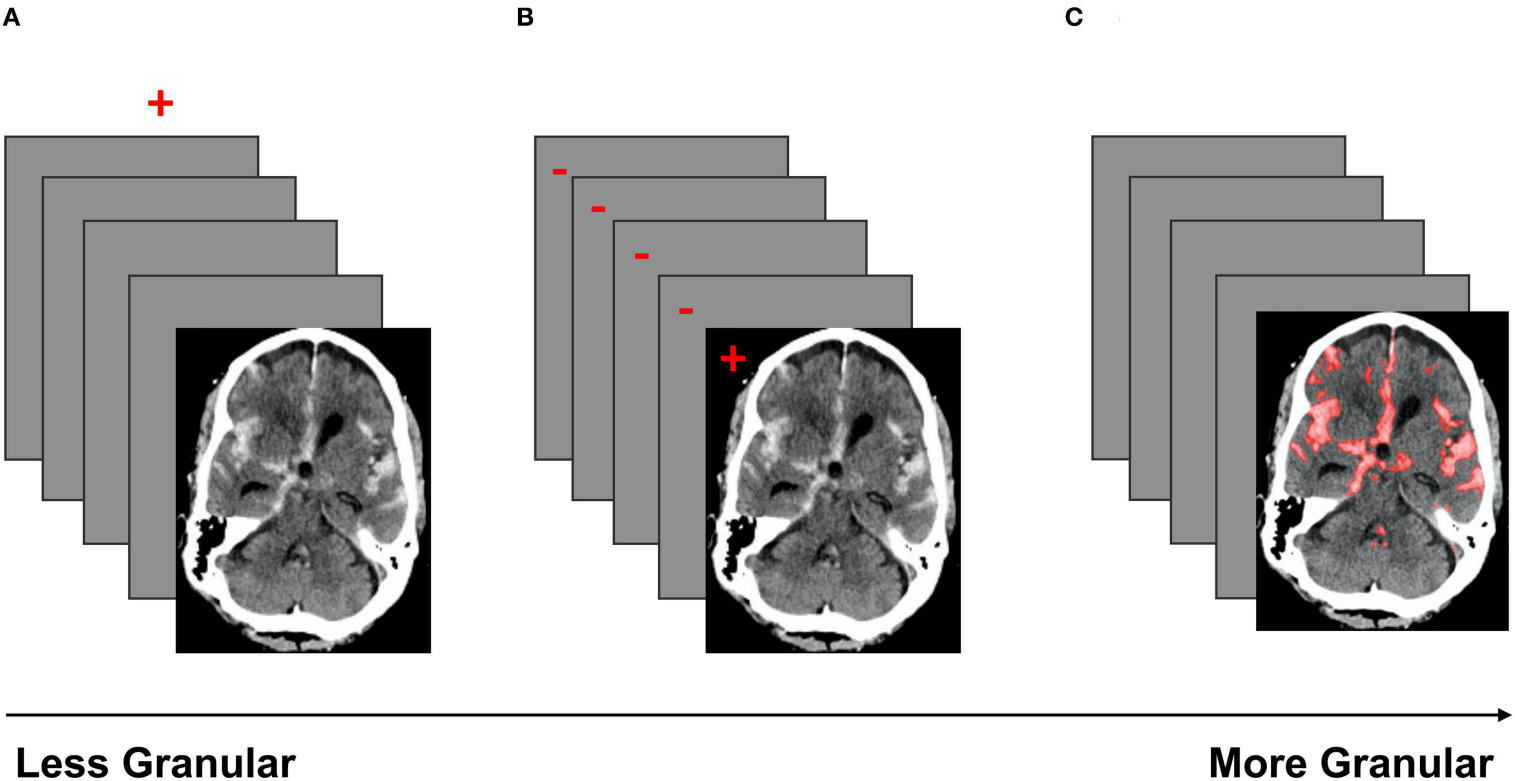
A schematic of three labeling strategies in order of increasing granularity. Red indicates the label(s). **(A)** Demonstrates examination labels, in which an entire exam is annotated as “positive” or “negative” for a given pathology. **(B)** Demonstrates image labels, where each image in a stack is annotated as “positive” or “negative” for a pathology. **(C)** Demonstrates pixel labels, where all pixels in the exam are labeled as “positive” or “negative” (29).

The coarsest data labeling strategy in medical imaging is examination-level labeling, which refers to categorization of each imaging exam based on the presence or absence of a specific type of imaging finding (Figure 4A). For example, a head CT exam typically contains a stack of 25–70 images, depending on the size of the head and the specific imaging protocol. If a CT exam has at least one instance of intracranial hemorrhage on at least one image, the entire exam would be annotated as “positive” for intracranial hemorrhage. The use of examination-level labels has several advantages, the primary being that these labels can generally be obtained with less time and cost, making it possible to amass larger datasets. In addition, examination-level labels can potentially be extracted from pre-existing clinical radiological reports by human reviewers without specialized training in radiology, or through the use of automated natural language processing algorithms. Examination-level labels represent the coarsest level of labeling and generally do not provide information on the location or size of abnormal findings.

Image-level annotations are an example of labels with an intermediate level of granularity. In this strategy, each image of an exam is annotated for the presence or absence of a specific type of imaging finding (Figure 4B). Unlike examination-level labels, image-level annotations provide some localization information, identifying the image(s) that contain instances of the finding of interest. Image-level labels provide significantly more information than examination-level labels, and can be used to improve the accuracy of exam-level predictions, as well as to provide coarse estimates of the locations and sizes of instances of the imaging finding of interest. However, image-level labels require significantly more cost and effort to obtain. Unlike examination labels, which are already present in clinical reports, image labels do not exist in the clinical setting and must be manually annotated by highly trained specialists.

Pixel-level annotation is the most granular labeling approach, and consists of the designation of each pixel in each image of the exam as positive or negative for the finding of interest. Pixel-level labels provide the densest information content (Figure 4C), and can be used to further improve the accuracy of exam-level predictions, as well as provide fine-grained localization information. Algorithms trained on high-quality pixel-level annotations have the potential to produce the strongest results because they are trained on the data with the densest information content. The primary disadvantage of this strategy is that pixel labels are costly and time-consuming to obtain, requiring manual delineation (“segmentation”) of the boundaries of each lesion or finding of interest. This is not a task that radiologists are accustomed to performing in a clinical setting, and is highly labor-intensive even with the use of optimized manual segmentation tools. Unlike examination-labeled datasets, the expansion of an existing pixel-labeled dataset may be prohibitively costly due to the labeling requirement. Regardless, algorithms trained on pixel labeled data can create stunning delineations of the boundaries of lesions, producing detailed location and size information that can be useful for clinical management decisions.

Bounding-box labels constitute a labeling approach that is intermediate between image-level and pixel-level labels. For example, Mask R-CNN (87) first detects an object and indicates its location by placing a bounding box that encloses all or part of the object, and subsequently performs a pixel-wise segmentation within the bounding box. However, hemorrhage is fluid and can take on nearly limitless morphologies, and is not well-suited to this approach. Mask R-CNN was designed primarily for the detection of discrete objects, and may be better suited to medical imaging applications that require identification of discrete pathological findings such as mass lesions.

These approaches illustrate several major strategies that have been used to label training data for deep learning algorithms for TBI image analysis. These concepts will appear again in later discussions of specific algorithms.

## Intracranial Hemorrhage Detection

**Figure 5:**
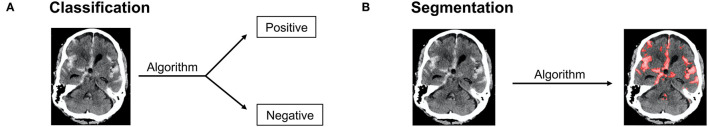
A schematic representation of **(A)** classification and **(B)** segmentation algorithmic outputs. In **(B)**, red regions indicate areas of acute intracranial hemorrhage designated by the algorithm (29).

Acute intracranial hemorrhage, which refers to acute bleeding within the confines of the cranial vault, is a key neuroimaging finding that determines the disposition of acute TBI patients from the ED. It is the only neuroimaging finding accepted by the U.S. FDA as a prognostic marker in acute TBI. Because neurosurgical interventions such as intracranial pressure monitor placement and craniotomy, including decompressive hemicraniectomy, must be performed emergently, algorithms that can reduce the time to diagnosis through the rapid detection and localization of acute intracranial hemorrhage could improve patient outcomes (106). A variety of deep learning approaches have been explored for intracranial hemorrhage detection. Some algorithms focus strictly on the classification of CT exams as positive or negative for intracranial hemorrhage, because this differentiation is a key determinant of immediate management steps (Figure 5A). A number of models that also attempt to predict different hemorrhagic subtypes have been reported. To further increase clinical usefulness and interpretability, algorithms may also perform segmentation, or detailed delineation of the hemorrhage boundaries (Figure 5B). Segmentation has the great advantage of allowing clinicians the opportunity to visually appreciate the algorithm's findings and to have an understanding of the extent/volume and location of the hemorrhage. Both segmentation and determination of the subtype of intracranial hemorrhage (intraventricular, intraparenchymal, subarachnoid, epidural, subdural, and petechial hemorrhage) are also critical for guiding immediate management decisions.

Table 1 summarizes prior algorithmic approaches for the detection and segmentation of acute intracranial hemorrhage in TBI. Although there is no formal distinction in the literature, we group the algorithms into two broad classes for illustrative purposes: triage algorithms and localization algorithms. Hereafter, approaches to image analysis in acute TBI will be described within the context of these two broad clinical objectives. Dataset sizes are described either in terms of number of CT exams or number of CT images. It is important to note that results from different papers cannot be compared head-to-head due to the use of different datasets and labeling strategies.

**Table 1:** Deep learning approaches for intracranial hemorrhage detection and segmentation on head CT.

**Publication**	**Model description**	**Training label type**	**Output type**	**Dataset size**	**Model performance**
Phong et al. (107)	Three models used: LeNet, GoogLeNet, Inception-ResNet	Examination labels	Hemorrhage detection	Train: 1,360 images Test: 340 images	LeNet accuracy: 0.997 GoogLeNet accuracy: 0.982 Inception-ResNet accuracy: 0.992
Prevedello et al. (23)	CNN	Examination labels	Hemorrhage, mass effect, hydrocephalus detection	Development: 246 exams Test: 130 exams	Sensitivity: 0.90 Specificity: 0.85 AUC: 0.91
Patel and Manniesing (108)	Convolutional neural network (CNN)	Image labels	Hemorrhage detection	Development: 150 exams Validation: 20 exams Test: 20 exams	Sensitivity: 0.87 Specificity: 0.97 Accuracy: 0.95
Majumdar et al. (109)	CNN	Pixel labels for hemorrhage subtypes	Hemorrhage detection	Development: 60 exams Validation: 5 exams Test: 69 exams	Sensitivity: 0.81 Specificity: 0.98
Grewal et al. (110)	Recurrent Attention DenseNet (RADnet)	Pixel labels for presence of hemorrhage	Hemorrhage detection	Development: 185 exams Validation: 67 exams Test: 77 exams	Accuracy: 0.818 Recall: 0.886 Precision: 0.813
Jnawali et al. (111)	3D-CNN	Examination labels	Hemorrhage detection	Development: 34,848 exams Validation: 2,000 exams Test: 3,509 exams	AUC: 0.87
Titano et al. (24)	3D-CNN modeled after ResNet-50	Examination labels	Urgent or non-urgent classification	Development: 37,236 exams Test: 180 images	AUC: 0.73 Accuracy: 0.56
Arbabshirani et al. (25)	CNN	Examination labels	“Routine” vs. “stat” classification	Development: 37,074 exams Test: 9,499 exams	AUC: 0.846
Chilamkurthy et al. (26)	CNN	Image labels	Detection of hemorrhage and subtypes	Development: 290,066 exams Validation: 21,095 exams Test: 491 exams	Overall hemorrhage AUC: 0.94 Intraparenchymal AUC: 0.95 Intraventricular AUC: 0.93 SDH AUC: 0.95 Extradural AUC: 0.97 SAH AUC: 0.96
Chang et al. (27)	Hybrid 3D/2D CNN	Pixel labels	Hemorrhage detection and segmentation	Development: 10,159 exams Test: 862 exams	Hemorrhage detection accuracy: 0.975 Hemorrhage detection AUC: 0.983 Hemorrhage detection sensitivity: 0.971 Hemorrhage detection specificity: 0.975
Ye et al. (112)	3D joint convolution and recurrent network (CNN-RNN)	Image labels	Detection of hemorrhage and subtypes	Hemorrhage development: 2,255 exams Hemorrhage validation: 282 exams Hemorrhage test: 299 cases Subtype development: 1,461 exams Subtype validation: 181 cases Subtype test: 194 cases	Hemorrhage classification: >= 0.98 across all metrics Subtype AUC: > 0.8 across all subtypes
Cho et al. (113)	Cascaded deep learning model	Pixel labels	Hemorrhage detection and segmentation	5.702 exams	Hemorrhage detection sensitivity: 0.979 Hemorrhage detection specificity: 0.988 Segmentation precision: 0.802 Segmentation recall: 0.822
Lee et al. (28)	ImageNet pretrained deep convolutional neural networks (DCNN)	Image labels	Classification of hemorrhage and subtypes; heatmap localization	Development: 704 exams Retrospective test: 200 exams Prospective test: 196 exams	Retrospective sensitivity: 0.98 Retrospective specificity: 0.95 Prospective sensitivity: 0.92 Prospective specificity: 0.95
Kuo et al. (29)	Patch-based fully convolutional neural network (PatchFCN)	Pixel labels	Classification of hemorrhage and subtypes; segmentation	Development: 4,396 exams Test: 200 exams	Hemorrhage classification AUC: 0.991 SDH AUC: 0.954 EDH AUC: 0.940 Contusion/ICH/TAI AUC: 0.934 SAH/IVH: 0.956 Dice coefficient: 0.75
Lee et al. (114)	“Kim-Monte Carlo algorithm,” an artificial neural network	Image labels with hemorrhage subtype	Hemorrhage detection and subtype classification	Training: 166 exams Validation: 84 exams	Overall AUC: 0.859 SDH/EDH accuracy: 0.534 SAH accuracy: 0.917 IPH/IVH accuracy: 0.60
Burduja et al. (115)	CNN-LSTM	Image labels with hemorrhage subtype	Hemorrhage detection and subtype classification	Training: 21,000 exams Validation: 744 exams Testing: 3,528 exams	Overall AUC: 0.9792 EDH AUC: 0.9414 IPH AUC: 0.9834 IVH AUC: 0.9866 SAH AUC: 0.9609 SDH AUC: 0.9451
Arab et al. (116)	Convolutional neural network with deep supervision (CNN-DS)	Pixel labels	Classification of hemorrhage; segmentation; volume quantification	Development: 45 exams Test: 10 exams	Dice coefficient: 0.84 Recall: 0.83
Sharrock et al. (117)	Convolutional neural networks with VNet framework	Pixel labels	Classification of hemorrhage; segmentation; volume quantification	Training: 100 exams Validation: 500 exams Testing: 12 exams	Mean dice coefficient: 0.911 Mean IVH dice coefficient: 0.911 Mean SDH dice coefficient: 0.879 Algorithm-human volume correlation: 0.98
Dhar et al. (118)	U-Net	Pixel labels	Classification of hemorrhage; segmentation; volume quantification	Training and cross-validation: 224 exams Testing: 84 exams	Dice coefficient: 0.90
Monteiro et al. (119)	CNN	Pixel labels	Classification of hemorrhage; segmentation; volume quantification	Training: 184 scans Testing: 655 exams	ICH external AUC: 0.83 IPH external AUC: 0.90 EAH external AUC: 0.80 IVH external AUC: 0.95 Overall mean dice coefficient: 0.36
Zhao et al. (120)	nnU-Net	Pixel labels	Classification of hemorrhage; segmentation; volume quantification	Training: 300 exams Validation: 80 exams	ICH dice coefficient: 0.92 IVH dice coefficient: 0.79

The publications in Table 1 are related to head CT, the standard neuroimaging study in the clinical management of acute TBI. In current clinical practice, brain MRI is only occasionally performed secondarily, as a problem-solving tool when neurological deficits persist throughout the hospital stay and/or are not adequately explained by head CT findings. While deep learning has been applied to brain MRI for other pathologies and disorders (121–123), there has been little development of such algorithms for MRI image recognition in acute TBI due to the relatively uncommon use of brain MRI in this clinical setting. MRI's current limited role in acute TBI also limits the quantity of available training data. Despite this, a small number of MRI hemorrhage detection algorithms have been proposed and are briefly discussed below for completeness (124–127).

### Triage Algorithms

Triage algorithms for analysis of clinical radiological exams are intended to expedite the interpretation of abnormal exams by bringing these to prompt attention by on-duty radiologists. One uniting feature of triage algorithms is their emphasis on exam-level classification; determination of detailed features such as location and size of abnormal findings is generally outside of the scope of algorithms in this category.

The performance requirement for a minimum viable triage algorithm is that its predictions are more accurate than random guessing. For algorithms intended for a more central role, e.g., augmenting the performance (accuracy) of radiologists through human/computer collaboration, the performance bar would likely be much higher. Still higher is the performance level that would be required for algorithms intended for use by non-radiologist clinicians for management decisions for acute TBI patients. It is important to note that these various performance bars are not defined by fixed metrics; rather, they are subjective in nature and depend on the intended “context of use” (128). Context of use is a key concept in FDA regulation of medical devices and other products, essentially providing a complete description of the intended clinical setting and manner of use of a medical product. In the case of algorithms for intracranial hemorrhage detection, context of use could include factors such as the expertise level of clinicians available to interpret acute head CT exams in a particular setting (e.g., emergency department physicians, neurosurgeons, general radiologists, or neuroradiologists), the typical turnaround time for CT interpretation (minutes to an hour), and how much the clinical management algorithm in a particular practice (e.g., the decision to admit or discharge) relies on head CT results.

Prior work has addressed the task of intracranial hemorrhage classification using a variety of technical approaches. Phong et al. (107) evaluated three different popular neural network architectures—LeNet (129), GoogLeNet (85), and Inception-ResNet (130)—for intracranial hemorrhage classification. All three models were widely used around the time of publication and had previously achieved state-of-the-art performance on benchmarked computer vision tasks. They were trained using exam-level labels, and achieved very strong performance on the intracranial hemorrhage classification task. However, the authors manually selected positive images to include in the dataset, and it is unclear whether negative images were also included. Patel and Manniesing (108) developed a convolutional neural network for intracranial hemorrhage classification using image-level labels and also achieved strong performance. Both Majumdar et al. (109) and Grewal et al. (110) proposed intracranial hemorrhage classification models using pixel level-labeling.

These studies collectively explored a variety of labeling strategies, from examination- to pixel-level labels. These papers primarily focused on the technical side of intracranial hemorrhage detection, exploring the effectiveness of various neural network architectures for hemorrhage detection and experimenting with technical hyperparameters. Each of these strategies demonstrated reasonable performances at minimum, with some even achieving accuracy metrics exceeding 0.90 (107, 108). However, a likely limitation is that each of these studies used a relatively small number of training and test cases (Table 1). Small training sets often lack adequate examples of the entire spectrum of imaging appearances of the pathology of interest, and the resulting models often fail to generalize to other data. In addition, performance results obtained on small test sets are often noisy.

In order to demonstrate clear clinical utility, it is important to demonstrate strong performance on larger datasets that are also representative of the intended population. Jnawali et al. (111) did this, collecting a large dataset of 40,367 head CTs to train and evaluate a three-dimensional convolutional neural network (3D-CNN) for intracranial hemorrhage classification. The model achieved an AUC of 0.87. Although these results were promising and obtained from experiments on large datasets, more work was needed to reach the performance bar needed for clinical use.

**Figure 6:**
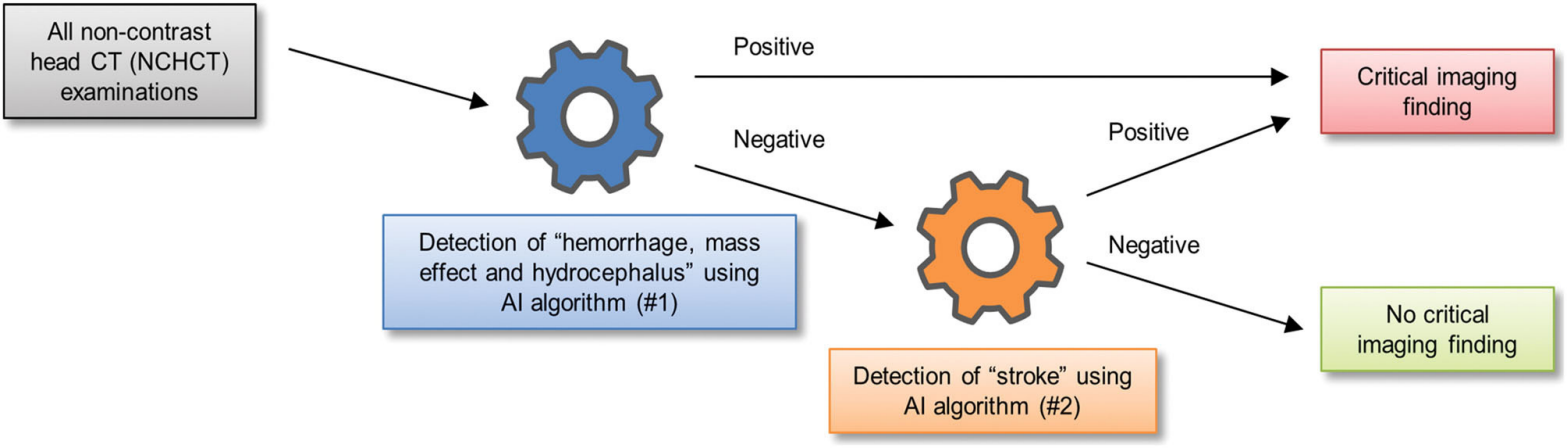
A schematic of one way in which two sequential algorithms can be integrated into triage workflow for exam classification (23).

One of the earliest works to study clinical integration was a triage algorithm proposed by Prevedello et al. (23) to draw attention to critical head CT exams for expedited evaluation by a radiologist. They developed two sequential deep learning algorithms, with the first dedicated to the detection of intracranial hemorrhage, mass effect, and hydrocephalus, and the second dedicated to detection of acute infarct. Each exam was read by the two algorithms serially and marked critical if either algorithm detected abnormalities (Figure 6). The first algorithm was trained on cases annotated with one of six possible examination-level labels: hemorrhage, mass effect, hydrocephalus, suspected acute infarct, encephalomalacia, or non-urgent/normal. For the first algorithm, the first three labels were considered “positive” and the last three were considered “negative.” During testing, the first algorithm classified cases as “positive” or “negative” for at least one of the first three imaging findings, demonstrating an AUC of 0.91. The second algorithm dedicated to acute infarct detection achieved an AUC of 0.81. Although hemorrhage, mass effect, and hydrocephalus were grouped for the purposes of algorithm evaluation, and performance on each distinct pathology was unknown, the study was important because it was one of the earliest to report a deep learning classification pipeline that could identify acute CT exams that contained one or more of a diverse collection of abnormal findings.

**Figure 7:**
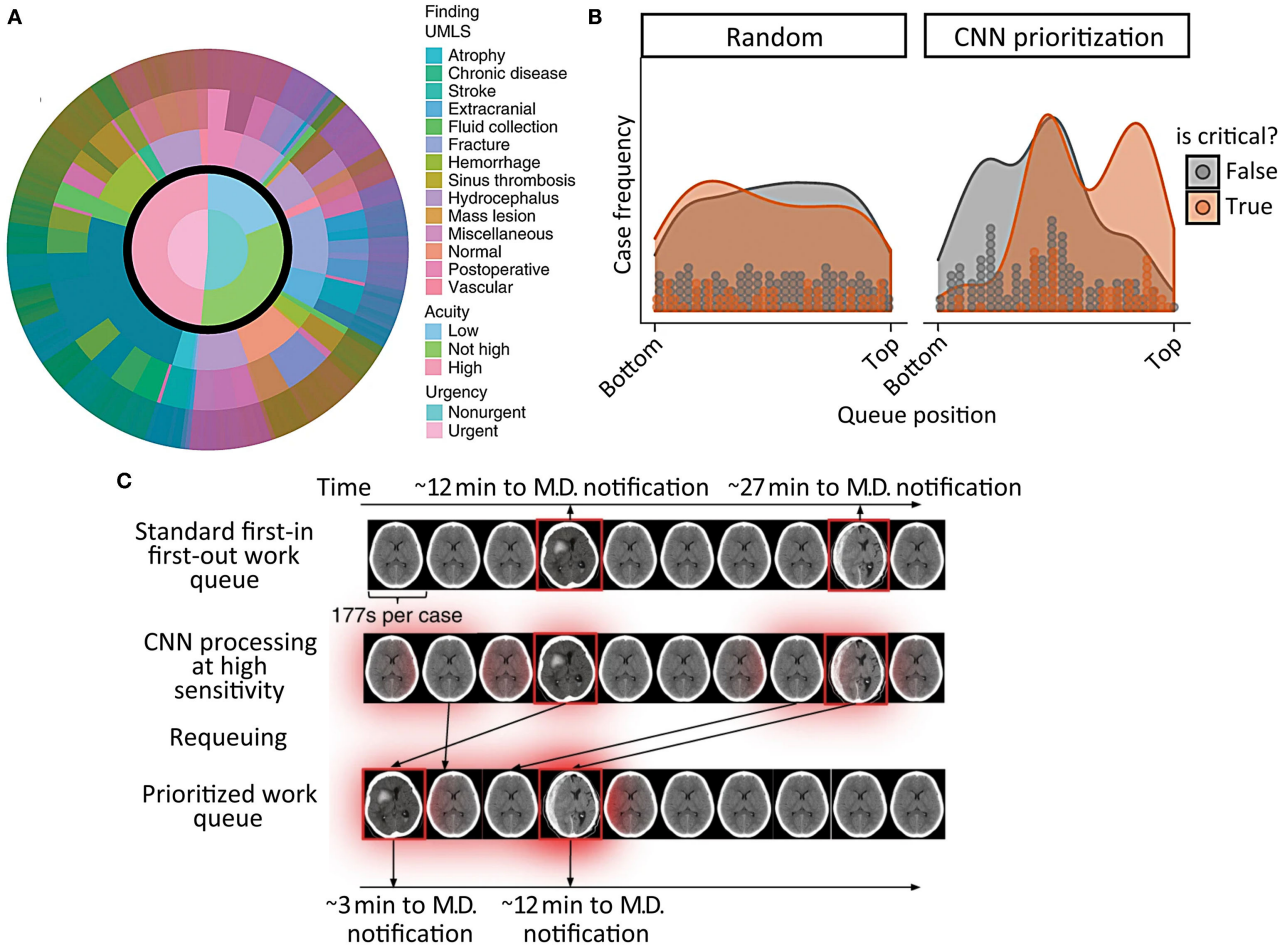
Incorporation of a reprioritization, or triage, algorithm into the radiological workflow in acute TBI (24). **(A)** Illustrates the broad range of pathological findings represented in the head CT training data, and how they were classified into non-urgent and urgent categories. **(B)** Shows the typical order in which critical (orange) and non-critical (gray) head CT exams would be interpreted by a radiologist before (left graph) and after (right graph) reprioritization by a deep learning algorithm. The gray and orange dots represent discrete CT exams, while the shaded regions are the smoothed exam frequency distributions. **(C)** Is a schematic representation of the algorithm's prioritization process.

Titano et al. (24) expanded upon this by proposing the integration of a deep learning system for radiological triage into a simulated clinical environment. These authors described an imaging triage system in which a 3-dimensional convolutional neural network (3D-CNN) re-ordered exams in the queue for radiological interpretation based on a much wider range of abnormal findings. The algorithm was designed to re-order exams so they would be reviewed on the basis of urgency rather than order of completion. The 3D-CNN was exposed to exams presenting with hundreds of head CT diagnoses based on the Universal Medical Language System (UMLS) concept universal identifiers, including intracranial hemorrhage, and each diagnosis was mapped to critical or non-critical categories depending on predetermined radiologist designations (Figure 7A). The 3D-CNN was trained on 37,236 head CT exams labeled at the exam level and produced exam-level classification outputs, demonstrating an AUC of 0.73 on 180 test images, when compared to the “gold-standard” labels of physician reviews of clinical reports. Exam re-prioritization resulted in a statistically significantly larger number of critical exams at the top of the queue, Figures 7B,C presents an example regarding how the algorithm identifies and reprioritizes exams in real time. This study was important because it demonstrated one of the earliest attempts to use a large dataset to learn a broad comprehensive range of pathologies. Because it involved a trial in a real-time simulated environment, it also allowed authors to quantify the speed of their algorithm in a hypothetical clinical setting and reach the conclusion that the algorithms were 150x faster than humans.

Expanding on this queue reordering study, Arbabshirani et al. (25) developed a deep convolutional neural network that was prospectively integrated into a radiological workflow for 3 months. The model's role was to re-prioritize exams from “routine” to “stat” if it detected an intracranial hemorrhage. Prior to integration, the model was trained on a large scale dataset of 37,084 exams with examination-level annotations and reported an AUC of 0.846 for intracranial hemorrhage classification. Following this initial development phase, the algorithm was integrated into the clinical workflow for 3 months. Over the course of that time period, the algorithm processed exams with an accuracy of 0.84. Exams prioritized as “stat” showed significantly reduced time to interpretation compared to “routine” exams, from a median time of 512–19 min. The Titano et al. and Arbabshirani et al. studies were two early papers that studied the impact of integration of deep learning triage algorithms into clinical settings.

**Figure 8:**
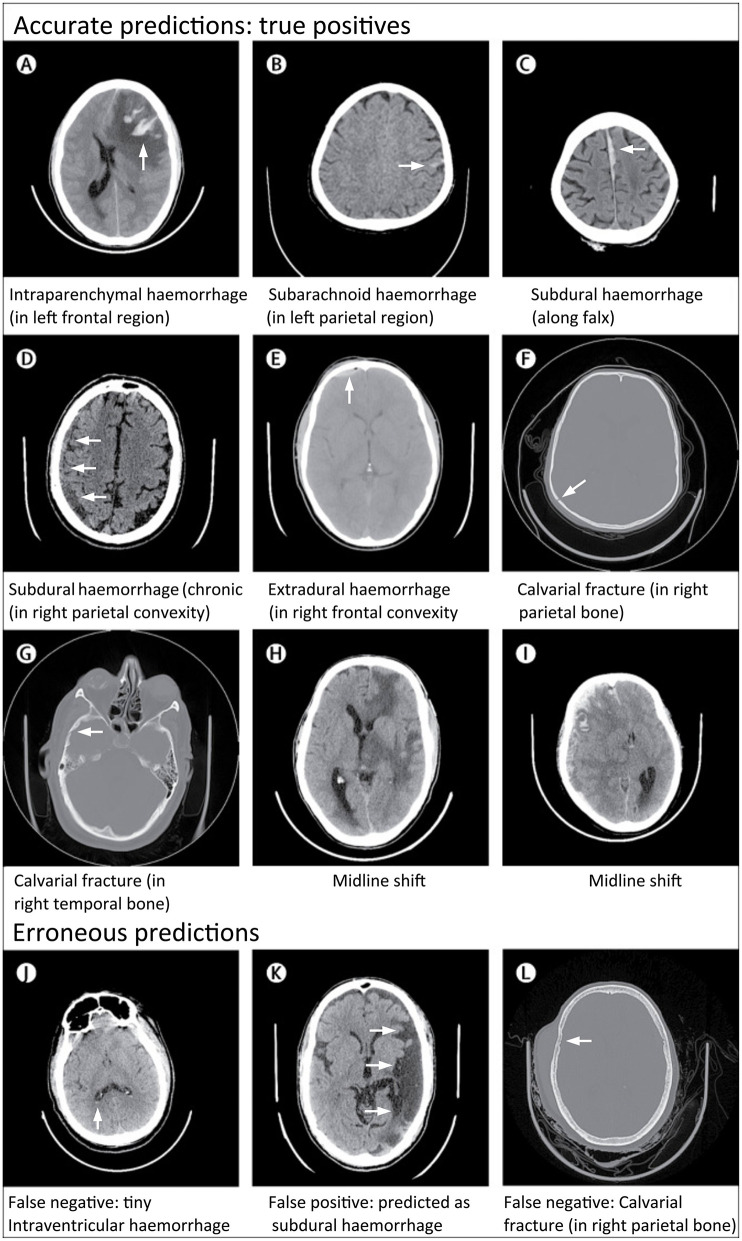
Examples of accurate and erroneous predictions of abnormalities on head CT in acute TBI patients by a deep learning algorithm (26). Although individual images are shown, the model classifies abnormalities at the head CT exam level. All images under Accurate Predictions **(A–I)** have arrows added to indicate the abnormal lesion. All images under Erroneous Predictions **(J–L)** have arrows added to indicate the erroneous lesion predictions.

Rather than perform broad critical and non-critical classifications of CT exams, Chilamkurthy et al. (26) focused instead on developing deep learning models that could identify specific head CT abnormalities, including intracranial hemorrhage subtypes (intraparenchymal, intraventricular, subdural, extradural, and subarachnoid), calvarial fractures, midline shift, and intracranial mass effect (Figure 8). The authors collected large datasets (290,055 CT exams) for training. The training and validation exams were labeled at the image level. The exams in the test set, referred to as “CQ500,” were each labeled by three highly experienced radiologists for the presence of intracranial hemorrhage and its subtypes, midline shifts, and skull fractures. Although exams were labeled at the image level, the model produced predictions at the examination level. The decision to leverage the more granular image training label likely helped achieve better exam-level predictions at test time.

This was the first study to evaluate model performance on individual pathologies, to use very large datasets (>100,000 CT exams), and to validate models on datasets representative of the patient population. Overall, the model performances across all pathologies were good, with the algorithm achieving an overall AUC of 0.94 for intracranial hemorrhage on CQ500. In Supplementary Materials, the authors indicated that they also trained a separate localization model to perform segmentation for intraparenchymal, subdural, and epidural hematomas, using a set of 1,706 images labeled at the pixel level according to hemorrhage subtype. Performance metrics and example images for these segmentations were unavailable at time of publication. The authors have also made the CQ500 test set publicly available to facilitate benchmarking of algorithms developed in the future.

Subsequently, triage algorithms with the capability to classify hemorrhages by subtype were described (112, 114, 115). Lee et al. demonstrated promising classification results with three hemorrhage subtype groupings (epidural/subdural, subarachnoid, and intraparenchymal/intraventricular hemorrhage) (114). As specific hemorrhage subtypes often influence management decisions, and also have different prognostic significance, later classification models were also proposed that identify each of the five subtypes individually (112, 115).

Aside from studies performed on CT, several studies have proposed intracranial hemorrhage detection algorithms on MRI. Most recently, Nael et al. (124) developed a set of deep CNNs which were each purposed to identify a different pathology on brain MRI, with intracranial hemorrhage as one such pathology. The AUC for intracranial hemorrhage detection on the internal and external test data was 0.90 and 0.83, respectively. Rudie et al. (125) proposed a neural network system trained to diagnose 35 different neurologic diseases on brain MRI, including 5 and 3 acute intracranial hemorrhage exams in the training and test sets, respectively. Previously, Al Okashi et al. (127) proposed an ensemble learning system for hemorrhage detection on brain MRI, but describes head CT images throughout the paper as brain MRI images. Le et al. (126) proposed R-FCN as a classification model for CT/MRI images to differentiate between different hemorrhagic subtypes. However, their methods and figures also use CT scans, such that the relevance to MRI is unclear. Overall, algorithms developed for MRI in acute TBI are currently limited.

Although studies have been performed on triage algorithms, limitations remain. It is reasonable to assume that lower performance standards for radiological triage algorithms are acceptable, as all exams are ultimately reviewed by trained human experts. However, this comes with the risk that missed critical findings, particularly those with more subtle abnormalities, may be reordered to the bottom of the queue, with an extended delay in review of these studies if there is over-reliance on the algorithm (131). This time delay could be exacerbated if additional exams are added to the queue in real time and receive higher priority. Explainability also remains important; if the rationale behind critical or non-critical classifications is opaque, it could be difficult for physicians to verify and trust algorithmic outputs (132, 133). “Black box” algorithms such as these also have the potential to lengthen overall readout times if radiologists spend extra time reviewing normal studies that were flagged as abnormal. In addition, lack of explainability may result in difficulty disregarding artifacts and other questionable findings in an exam that has been flagged as abnormal. This could lead to overdiagnosis, a difficult problem noted in early studies of the use of CAD in screening mammography (134). Finally, the identification of hemorrhage locations and sizes is also critical in order to inform neurosurgical decisions.

### Localization Algorithms

Localization algorithms are trained to predict the locations of abnormal imaging findings. In these algorithms, more general exam-level classifications are also usually derived indicating the overall presence or absence of the finding of interest anywhere on the exam. Localization algorithms mitigate the explainability problem, as their outputs specify lesion locations, such as by direct annotation of the original CT images. They also leverage the quantitative nature of algorithms, as detailed localizations have the potential to produce volumetric outputs, which are difficult and time-consuming for humans to measure accurately. Although development of localization algorithms usually require the denser, pixel-level labels, which are highly time-consuming and costly to obtain, they have the potential for providing information that is useful for clinical management decisions as well as outcome prediction.

Chang et al. (27) reported an approach to the task of localizing acute intracranial hemorrhage, using a hybrid 3D/2D convolutional neural network for exam-level classification and hemorrhage segmentation derived from Mask R-CNN (135). They collected a large training set annotated with bounding-box labels around areas of hemorrhage that were verified by a board-certified radiologist. The model demonstrated excellent classification performance, achieving an AUC of 0.981 on the test set. To incorporate localization information, semi-automated pixel labels were produced for hematoma regions using level set segmentation (136). The Dice coefficients for manual and model segmentations for intraparenchymal, epidural/subdural, and subarachnoid hematomas were strong, with decreasing Dice indices in that order. Not surprisingly, the Dice coefficient for subarachnoid hemorrhage was the smallest, as this type of hemorrhage is the most difficult to segment accurately since the bleeds tend to be amorphous with poorly-defined boundaries. Hemorrhage volumes based on manual vs. model segmentations were also computed, with very strong Pearson correlation coefficients exceeding 0.95 for all three hemorrhage subtype categories. Taking a similar approach to the localization problem, Cho et al. (113) collected a pixel labeled dataset and proposed a cascaded deep learning for hemorrhage subtype identification and segmentation. The model also achieved strong performances, with a classification accuracy of 0.979 and segmentation precision of 0.802. These works were promising as they demonstrated excellent performance in classification and localization of hemorrhage, although segmentation examples for more subtle or complex hemorrhages were not available at the time of publication. In addition, the object detection and region processing requirements associated with Mask R-CNN are challenges, since hemorrhage is not discrete but is fluid and takes on highly variable morphologies that are not as well-suited to bounding-box labels. In addition, when pixel labels are obtained with the assistance of a level set algorithm rather than through manual labeling, the segmentations could be subject to the biases of the level-set algorithm assumptions, carrying with it the possibility of reduced label quality particularly for hemorrhage that is subtle, diffuse, and/or amorphous.

**Figure 9:**
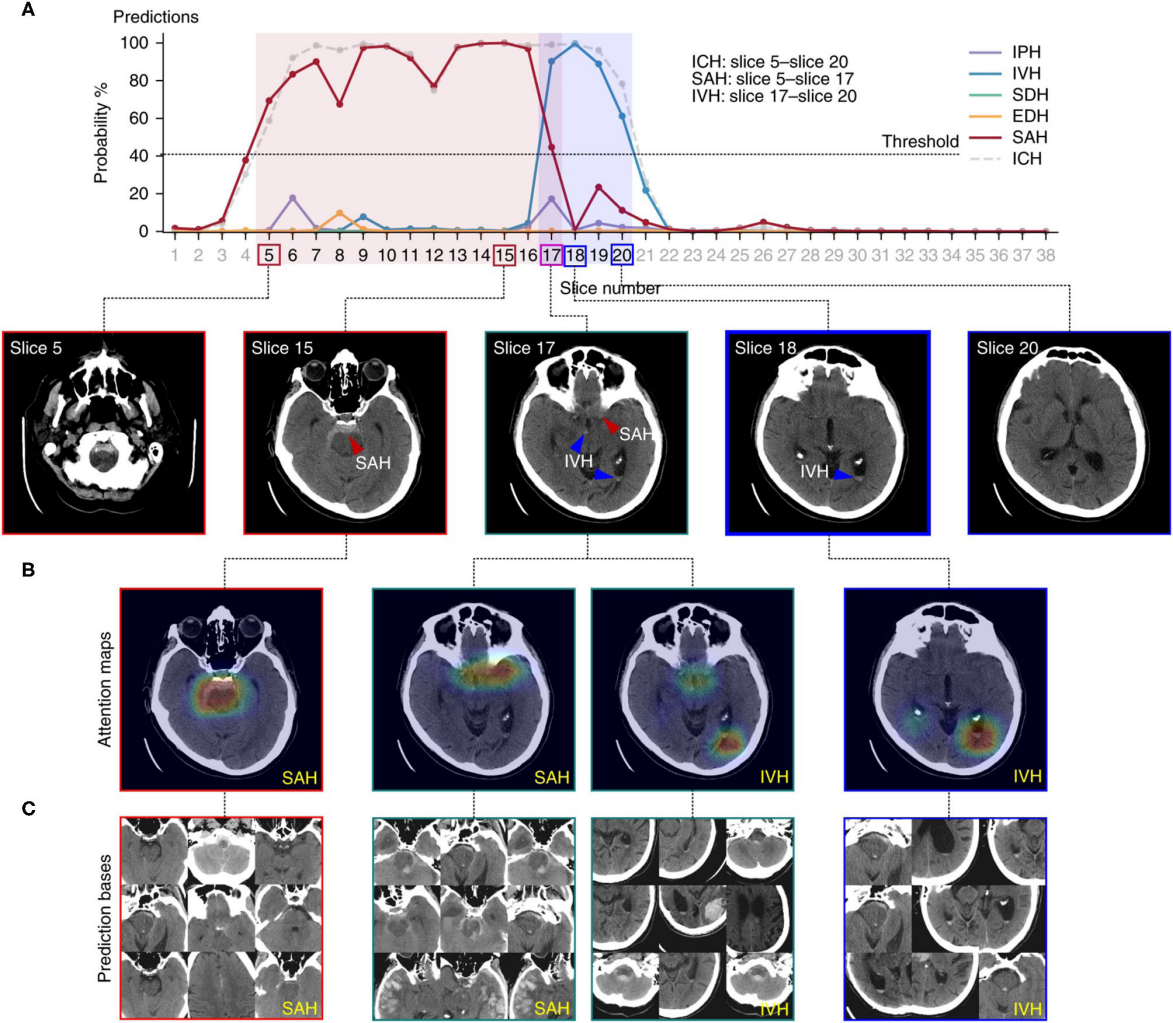
An example of a deep learning algorithm to localize intracranial hemorrhage and predict its subtype (28). Figure depicts the algorithmic output for a single head CT exam. **(A)** Demonstrates the probability determined by the algorithm for presence of each subtype of intracranial hemorrhage on each image. A 40% probability was designated as the minimum probability threshold to indicate the presence of a hemorrhage subtype on an image. The legend to the right shows the intracranial hemorrhage subtype that corresponds to each color. The boxes around the slice numbers indicate the example slices shown in the row of images below the graph, with the colors of the boxes indicating hemorrhage subtype(s) present on each image. Colored arrows on the images indicate the general prediction location and hemorrhage subtype. In **(B)**, a probabilistic heatmap is superimposed on the brain to indicate a more specific region of prediction. **(C)** Displays prediction bases, which are the most relevant training images for specific hemorrhage subtypes. These can be examined by human practitioners to gain insight into the main drivers, or “rationale,” behind the algorithm's predictions, thereby increasing explainability.

Lee et al. (28) used a different approach to the localization task. Employing only image-level labels for training, the algorithm predicted locations of abnormal findings using image-level “heatmaps.” The authors collected a training set as well as prospective and retrospective test sets. Each of the images was labeled according to the hemorrhagic subtype present, including intraparenchymal, intraventricular, subdural, epidural, and subarachnoid hematomas. Using only image level labels, the authors developed an understandable deep learning algorithm that detects the presence of acute intracranial hemorrhage and identifies it as one of five possible hemorrhage subtypes. The algorithm achieved excellent performance with an AUC of 0.99 on the retrospective set and 0.96 on the prospective set. In order to visualize the locations of predicted lesions, the system generated probability heatmaps that used color to highlight high-probability pixels for hemorrhage, along with the suspected hemorrhage subtype (Figure 9). The input-output pairing in this study is relatively uncommon. As seen earlier, the most common strategies produce predictions with the same degree of granularity as provided by the training labels. This system, in contrast, predicts the general location of hemorrhage on each image, despite training data consisting of only image-level labels. This localization allows clinicians to directly inspect and verify the predictions, avoiding the “black box” problem, and enabling clinicians to independently evaluate and potentially understand the rationale behind the algorithm's predictions, including possible reasons for error.

**Figure 10:**
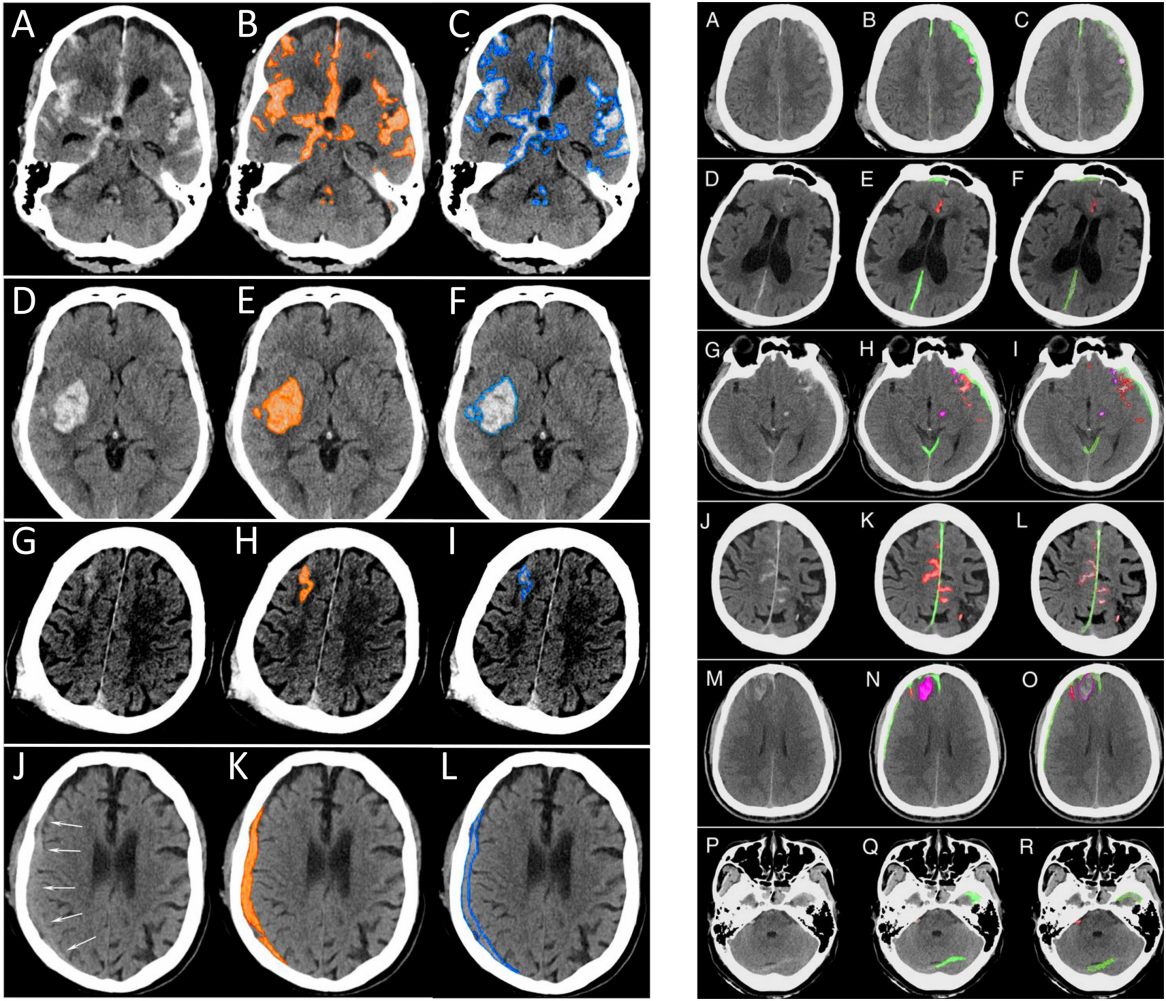
Examples of intracranial hemorrhage detection, classification, and segmentation by a convolutional neural network (CNN) (29). (Left) Binary segmentations, in which the model indicates the presence or absence of intracranial hemorrhage only. **(A,D,G,J)**. The first column shows the original head CT images. (B,E,H,K) The middle column shows the same images with orange shading of pixel-level probabilities >0.5 for intracranial hemorrhage as determined by the CNN. **(C,F,I,L)** The third column shows the original images with a blue outline drawn by an expert neuroradiologist around all areas of intracranial hemorrhage. (Right) Multiclass segmentations, in which the model not only detects intracranial hemorrhage but additionally indicates the hemorrhage subtype. **(A,D,G,J,M,P)** The first column shows original head CT images. **(B,E,H,K,N,Q)** The second column shows the algorithm's predictions. Each subtype is indicated by a different color, where subdural hematoma is green, brain contusion is purple, and subarachnoid hemorrhage is red. **(C,F,I,L,O,R)** The third column shows the “ground truth” labels drawn by expert neuroradiologists.

Kuo et al. (29) used a different approach, training a deep learning model on CT exams with hemorrhage labeled at the pixel level. The authors developed a patch-based fully convolutional neural network, which was optimized to perform joint classification and segmentation of intracranial hemorrhage. The algorithm had an identical input-output pairing (i.e., input training data and output prediction data with identical granularity of the labels). Training data consisted of 4,396 pixel-labeled CT exams. Rather than outputting heatmaps with diffuse boundaries, as seen with the Lee et al. study, this algorithm's segmentations provide high-resolution localization information (Figure 10, left panel). The classification performance was benchmarked against four radiologists on an independent evaluation set, in which the network outperformed two of the four radiologists, achieving an AUC of 0.991 for the classification task and a Dice coefficient of 0.75 for the segmentation task. An exploratory multiclass study was also conducted, in which the model identified and segmented different hemorrhagic subtypes. Segmentation visualizations from the multiclass study were also shown (Figure 10, right panel). This study was important because it included a broad selection of visualization examples, demonstrating detailed intracranial hemorrhage segmentations of intracranial hemorrhage and its subtypes. However, this approach requires a dataset annotated at the pixel level, which is highly time-consuming, expensive to obtain because it requires highly-trained human experts, and difficult to scale. Despite this, the study demonstrated the potential for deep learning algorithms to achieve expert-level classification performance and excellent segmentation, provided densely annotated data are available.

More recently, there has been interest in the use of algorithms to quantify intracranial hemorrhage volumes. Although lesion volumes can hold important prognostic significance and have been established to correlate with mortality and functional outcome (137, 138), they are difficult to obtain accurately. Hematoma volumes are currently estimated using the ABC/2 method; however, this form of measurement assumes an elliptical hematoma and is not suitable for most types of acute intracranial hemorrhage in TBI (139). As segmentation models began to show stronger performance, interest in volumetric analysis of intracranial hemorrhage increased. Some studies focused only on computing total intracranial hemorrhage (116, 118) while others computed separate volumetric outputs for different hemorrhage subtypes (117, 119, 120). While the multiclass volumetric studies demonstrated promising results, many grouped multiple pathoanatomic lesion subtypes into the same category, although different subtypes often require very different clinical management steps. Other studies either did not report or combined results for lesion subtypes that are difficult to segment or detect, such as subarachnoid or petechial hemorrhages. Finally, many studies used small tests due to the difficulty of obtaining ground truth manual segmentations, which raises the possibility of poor generalizability. While initial results are promising, more work is needed to develop algorithms that can perform volumetric analysis in an accurate, reproducible, and comprehensive way.

## Skull Fractures

CT is the preferred imaging modality for the diagnosis of acute skull and facial fractures. Since non-displaced skull fractures without intracranial hemorrhage heal without intervention, automated skull fracture detection has received less attention than intracranial hemorrhage detection and is a relatively less-explored area. Automated skull fracture detection is challenging since the typical non-displaced skull fracture is a tiny feature (often <1 mm in size) on any single CT image, and can only be differentiated from normal venous channels and sutures (140, 141) by its appearance over multiple contiguous images. Most prior attempts at skull fracture detection have used traditional morphological processing techniques. Shao and Zhao (142) used region-growing and boundary-tracing to define the skull, and optimal thresholding techniques to detect the fractures. Zaki et al. (143) used fuzzy c-means (FCM) clustering and line tracing to localize the fractures. Yamada et al. (144) used a black-hat transformation (technique to highlight dark objects of interest in a bright background) to identify the fractures. However, none of these approaches have made a transition into clinical use.

In more recent years, researchers have applied deep learning strategies to approach skull fracture detection. Chilamkurthy et al. (26) used an algorithm trained on image-level labels to perform exam-level detection of calvarial fractures, achieving an AUC of 0.96 on the CQ500 test set. Heimer et al. (145) trained a collection of deep learning models to identify skull fractures on postmortem computed tomography (PMCT) exams, achieving an average AUC of 0.895 on a test set of 150 cases, half of which contained skull fracture. Ning et al. (146), developed an attention-based multi-scale architecture (AMT-ResNet) for skull fracture detection, achieving 0.903 accuracy and 0.922 recall on a test set of 1,236 images with and 1,300 without skull fracture. Kuang et al. (147) proposed another neural network architecture (Skull R-CNN) based on modification of Faster R-CNN (148) to improve detection of smaller objects, achieving a test AP of 0.60 on a small test set of 10 cases. However, studies of deep learning models for skull fracture detection remain preliminary, with relatively small datasets and no studies to date that have demonstrated performance level or capabilities that would be consistent with practical utility in a clinical setting.

## Intracranial Mass Effect

Intracranial mass effect occurs when space-occupying lesions (hematomas, tumors, enlarged ventricles, vasogenic, or cytotoxic edema) result in significant displacement of a portion of the brain, or when brain swelling due to diffuse insults such as encephalitis or hypoxic-ischemic injury results in elevated intracranial pressure. Severe intracranial mass effect is an emergency and may require medication, extraventricular drain placement, or craniectomy to avoid loss of brain tissue and/or reduced perfusion of the brain. Intracranial mass effect in the setting of acute closed head injury usually takes the form of midline shift and/or downward cerebral herniation due to subdural or epidural hematoma, or large brain contusions that often develop surrounding vasogenic edema in the subacute stage. Despite the clinical importance, however, works studying the automated detection of intracranial mass effects are limited.

The first algorithm in the previously described double-algorithm framework of Prevedello et al. (23) detected acute intracranial hemorrhage, intracranial mass effect, and hydrocephalus, achieving an AUC of 0.91. However, the three abnormalities were ordered in a hierarchical structure (acute hemorrhage > mass effect > hydrocephalus), such that each CT exam was labeled with only the highest-ranked category during training, and the reported accuracy was for all three abnormalities collectively. Therefore, the model accuracy for intracranial mass effect alone was unknown. In addition, the representation of intracranial mass effect in the datasets was low: only 13 of the 246 training/validation cases and 5 of the 130 test cases were “positive” for intracranial mass effect. A practical problem in developing algorithms in this area is that intracranial mass effect is a relatively uncommon CT finding compared to intracranial hemorrhage, making it more difficult to collect the quantity of data needed to achieve strong performance.

As described earlier, Chilamkurthy et al. (26) developed a deep learning algorithm to simultaneously detect intracranial mass effects including midline shift, demonstrating an AUC of 0.92 on the CQ500 test set. The model was trained with 699 CT exams (320 exams positive for mass effect), for which each image was labeled by a radiologist for the presence or absence of intracranial mass effect. Finally, Monteiro et al. (119) developed a deep learning algorithm to segment vasogenic edema surrounding hemorrhagic contusions.

## Stroke

Ischemic stroke, which makes up 85% of all stroke incidents, results when blood flow is hindered due to arterial blockage and can cause permanent brain tissue damage (149). TBI has been previously identified as a risk factor for ischemic stroke (150–152); head injuries may lead to cerebrovascular damage through vascular shearing mechanisms, as well as compression of the anterior cerebral arteries due to subfalcine herniation or posterior cerebral arteries due to downward cerebral herniation. Acute stroke incidence in acute TBI patients is low, affecting ~2.5% of moderate and severe TBI patients (45). As with acute intracranial hemorrhage and intracranial mass effect in acute TBI, rapid detection and treatment of acute stroke is needed to achieve favorable outcomes (153, 154). Although a comprehensive discussion of machine learning techniques for detection of acute stroke is beyond the scope of this targeted review, a brief discussion of algorithmic approaches for automated stroke detection follows.

There is a considerable body of work focusing on the algorithmic detection and segmentation of acute ischemic stroke (92, 103, 155–161), although not specifically within the context of TBI. As with computational approaches to image recognition in acute TBI, older studies used rule-based and traditional machine learning techniques (162, 163), and descriptions of deep learning approaches to this problem have appeared only in the past several years. Unlike acute TBI, acute stroke is commonly diagnosed using a number of different imaging modalities and protocols, including non-contrast head CT, CT perfusion of the brain, CT angiography of the brain and neck, and MRI. Wang et al. (164), one of the earliest studies using deep learning in this area, reported a deep symmetry CNN that achieved promising stroke segmentation results on brain MRI, though using a very small sample size of eight patients. Subsequently, Zhang et al. (103) reported a CNN that performed acute stroke segmentation on diffusion-weighted imaging (DWI) with a Dice coefficient of 0.79, using training and test sets of 90 subjects each. Recently, Liu et al. (92) reported and publicly released a deep learning model that segmented acute ischemic stroke lesions on diffusion-weighted imaging (DWI) with a Dice coefficient of 0.76, similar to interrater agreement among human experts. The model was tested on a dataset of 2,348 DWI images, apparently the largest test set reported to date for this application. The model was also evaluated on an external dataset for generalization, and generally outperformed its peer models. Aside from applications in diagnosis of acute stroke on imaging, deep learning algorithms have also been proposed to predict stroke expansion across time (165–167) and identify ischemic stroke subtype (168, 169). As in acute TBI, these could eventually be useful for improved prognostic assessments and more personalized clinical management recommendations.

## Challenges and Future Directions

Despite major recent advances in computer algorithms for analysis of images in acute TBI, there remain significant challenges and opportunities for expanded use of these algorithms in clinical settings. Firstly, while algorithms for intracranial hemorrhage detection have been developed with accuracy levels acceptable for triage, more widespread clinical use of these algorithms is unlikely until algorithms for the reliable detection of other important abnormalities such as intracranial mass effect, acute and subacute infarct (including both large-territory and small-vessel infarcts and hypoxic/ischemic injury), bony fractures, and edema have been developed. While there have been algorithms developed for the above abnormalities, they remain limited and less thoroughly explored. In addition, while a limited number of localization algorithms for acute intracranial hemorrhage have been demonstrated, the need for localization and explainability applies to other pathologies such as intracranial mass effect, acute infarct, and fractures. Algorithms need to reliably identify all intracranial hemorrhage and skull fractures, including bilateral or subtle abnormalities, as these are important for surgical management decisions in TBI, and the failure to do so can lead to devastating consequences (170, 171). High-resolution pixel-level localization also raises the possibility of objective quantitative measurements of abnormal features, which could be used to improve clinical practice guidelines in the pursuit of precision and evidence-based medicine.

Generalization of strong model performance, to institutions other than those that provided training data for the model, is also a challenge. When algorithms trained on data from one institution are applied to data from another institution, the performance degrades to varying degrees. This is attributable to differences in hardware and other technical parameters that greatly affect the appearance of the images. For an algorithm to be widely deployed, it must remain robust to variations in image appearance across institutions, as well as with ongoing technical innovations in CT scanner hardware and image post-processing techniques over time.

Aside from challenges with algorithm development, another impediment to progress in the field is the lack of dataset standardization. Research teams often collect their own datasets, which can vary by important factors such as dataset size and degree to which it accurately reflects the patient population for intended use. This lack of standardization makes it challenging to compare different models head-to-head, and can also make it difficult to measure progress in the field through rigorous benchmarking. Although CQ500 (26) was a publicly released dataset, several factors hinder more widespread use. Because CQ500 was released without the accompanying training set, research teams would still be required to collect their own training data, which would differ from CQ500 due to variations in technical hardware, scanning protocols, and image post processing techniques. Large, high-quality datasets are usually not released, due to the sensitivity of medical data and institutional restrictions.

In the clinical setting, the development of accurate algorithms for medical image recognition also opens the potential for improved prognostic models, improved metrics for monitoring disease progression, and more specific patient selection criteria for clinical research studies. Outcome studies that include imaging findings (in addition to demographic and clinical variables) as predictors often take into account the presence or absence of certain imaging abnormalities, as it is challenging to incorporate granular imaging information in a scalable and reproducible way (172, 173). The qualitative presence or absence of abnormalities can be extracted from clinical radiology reports, or from an additional dedicated radiological interpretation performed for research purposes. However, more sophisticated TBI classification schemes are a critical need in order to develop effective therapies for acute TBI (34). In TBI and other disorders, patient outcomes often vary widely based on more granular information such as number, size, subtype, and location of abnormalities, that is available but not generally accessible from imaging studies on a large scale. Models that can quantify intracranial hemorrhage volumes and predict intracranial hemorrhage subtypes have been and are continuing to be studied for this purpose (118–120). In addition, the quantitative analysis of other features, including brain parenchyma CT densities (174, 175), regional brain volumes and atrophy (176), DTI (53, 54), and deep learning-based anatomical segmentation on MRI (177, 178) will also have a future role in improved clinical management and prognostication.

## Relevance to Precision and Evidence-Based Medicine

Precision medicine aims to “transform healthcare through use of advanced computing tools to aggregate, integrate and analyze vast amounts of data… to better understand diseases and develop more precise diagnostics, therapeutics and prevention.” (179). The capability to extract clinically relevant features from medical images has the potential to enable integration of imaging into precision medicine by transforming medical imaging into an increasingly quantitative and objective science.

For example, the FDA does not consider radiological interpretation, even by highly-trained human experts, to meet its definition of a “biomarker” as a “defined characteristic that is measured as an indicator of normal biological processes, pathogenic processes, or biological responses to an exposure or intervention...” (128). Biomarkers must be quantitative, objective and reproducible measures (180–183). Biomarkers such as blood test results and genomic data are reproducible and quantitative in nature. In contrast, human interpretation of medical images, even when performed by trained experts, remains subjective, making it difficult to aggregate their content into precision medicine. Indeed, the FDA regards human interpretation of imaging as a “clinical outcome assessment” (COA) (128), defined as a test with results that may vary considerably due to differences in subjective interpretation by human observers, as a result of differences in their judgment and experience. Thus, the use of automated methods to extract quantitative information from medical images has the potential to accelerate the development of FDA-qualified imaging biomarker tests (184, 185), which are currently nearly non-existent in TBI. This in turn could for the first time enable aggregation of imaging biomarkers with clinical, genomic and other patient data across centers to answer important questions regarding prognosis and best treatment practices in TBI and other neurological diseases (186).

Along similar lines, despite the increasing use of clinical practice guidelines to encourage uniformity in clinical management, clinical practice guidelines that include information from radiological images continue to rely almost exclusively on human interpretation. Evidence-based medicine involves the “conscientious, explicit, and judicious use of current best evidence in making decisions about the care of individual patients” and relies on the integration of “individual clinical expertise with the best available external clinical evidence from systematic research” (187, 188). As artificial intelligence algorithms for TBI image analysis mature, there is potential to improve practice guidelines using more reproducible data extracted from images. For example, a “midline shift” of 5 mm or more correlates with worse patient outcome (189, 190) and is one criterion used in the decision to perform decompressive craniectomy (191). Automated methods have the potential to promote the development of more advanced, granular metrics of intracranial mass effect that lead to practice guidelines that are better tailored to individual patients.

## Author Contributions

Both authors listed have made a substantial, direct, and intellectual contribution to the work and approved it for publication.

## Conflict of Interest

EY is an author of USPTO No. 62/269,778, Interpretation and quantification of emergency features on head computed tomography, and PCT Patent Application No. PCT/US2020/042811, Expert-level detection of acute intracranial hemorrhage on head CT scans, both assigned to Regents of the University of California. The remaining author declares that the research was conducted in the absence of any commercial or financial relationships that could be construed as a potential conflict of interest.

## Publisher's Note

All claims expressed in this article are solely those of the authors and do not necessarily represent those of their affiliated organizations, or those of the publisher, the editors and the reviewers. Any product that may be evaluated in this article, or claim that may be made by its manufacturer, is not guaranteed or endorsed by the publisher.
